# The Book World of Medicine and Science

**Published:** 1898-09-17

**Authors:** 


### The Book World of Medicine and Science.

Tropical Diseases: A Manual of the Diseases of Warm Climates. By Patrick Manson, M.D., LL.D. (Aberd.) With 88 illustrations and two coloured plates. (London: Cassell and Co. 1898. Price 10s. 6d.)

In its plan and in its size this work is strictly a manual, and it is stated in the preface that the reason for its production is that the exigencies of travel and of tropical life are, as a rule, incompatible with big volumes and large libraries. Yet, in reading it, we cannot but feel that this is not its only *raison d'être*. No doubt it supplies a want, but it is by no means a book written to meet a mere commercial demand. Every page shows that the author's knowledge is derived from personal experience, and that he speaks as one who has something to say. Nothing can be more striking than the very large share taken by parasitic organisms in the production of tropical diseases, and on this subject Dr. Manson writes as a master. In his selection of topics the author has very properly placed upon himself certain limitations. If by the term "tropical diseases" were meant only such ailments as are peculiar to and entirely confined to the tropics, then, as he says, half a-dozen pages might have sufficed for their description; while on the other hand if tropical diseases be held to include all ailments occurring in the tropics, a work professing to deal with them would require to cover almost the entire range of medicine, for the diseases of temperate climes occur also in the tropics. But Dr. Manson employs the term "tropical" in a meteorological rather than in a geographical sense, meaning by it sustained high temperature, and by "tropical diseases" he means diseases which occur only or especially in warm climates. Here, however, certain qualifications must be insisted on which bring us to the key note of much that the book has to say. The climate of tropical countries does not induce peculiar diseases by dint of the direct effects of the all-pervading heat, so much as by virtue of the fact that the commonly prevailing disease-germs are more or less peculiar to hot climates. Whether in temperate climes or in the tropics, whether in Europeans or in the natives of hot countries, disease properly so called, at any rate acute disease, is in all countries due to specific causes, and the difference between the diseases of temperate and of tropical climates is to a large extent dependent on the different specific causes of disease to which men in different climatic zones are most exposed. Again, the predominance of the specific causes of tropical diseases depends not merely upon heat but upon other co-operating conditions, more especially upon the fact that in many cases the "germ" is not carried directly from man to man, but requires the intervention of an intermediate host. Thus it happens that the effect of climate in favouring the spread of this or that disorder may depend not on its offering conditions favourable or otherwise to the growth of the parasite or germ, but to the degree of encouragement it may offer to the development of those forms of life which are essential to the transport of these germs from host to host. Dr. Manson very properly devotes a considerable amount of space to a consideration of fevers, among which he includes heat-stroke. The description of the malaria parasite and its various phases is very good, and if one could but feel sure about the mode by which the germ enters the body of the patient it would afford a reasonable explanation of the phenomena met with. One cannot avoid the feeling, however, that even though the whole sequence of events within the body of both man and mosquito be accepted as proved, there is a gap between the mosquito and the man. A good description is given of dysentery in its several forms, and the water origin of the disease in the vast majority of dysentery epidemics is insisted on. A large number of diseases are mentioned which will be quite unfamiliar to those who practise in temperate climates, and the very fact that such diseases exist and apparently claim a not inconsiderable number of victims shows the necessity for special study of such ailments by those who intend to practise in hot countries. For such no manual could be better than the one before us. While it is concise, it is quite full enough for practical purposes, and while the author speaks as one whose voice should be listened to, he gives fair and sufficient prominence to the views of others.

The Extra Pharmacopœia, Revised in Accordance with the "British Pharmacopœia," 1898. By William Martindale, F.L.S., F.C.S. With Serotherapy, Organotherapy, Medical References, and a Therapeutic Index, by W. Winn Westcott, M.B.Lond. Ninth edition. (London: H. K. Lewis. 1898. Price 10s. 6d.)

We are glad to welcome a new edition of this most handy and popular little book. It is many years now since it first appeared, and stepped at once into favour as a useful and reliable companion on the prescriber's desk, and by dint of keeping much to the old form, and by retaining in full its old characteristics of accuracy and fulness of information it seems likely to retain its place for long. What the physician wants in his daily life is not a mere official pharmacopœia, a list of drugs which have stood the test of time and criticism; what he requires is a list of all sorts and kinds of drugs that happen just at present to be in common use, with information as to their properties, their doses, and their mode of administration, and with references to literature on the subject. This is just what Mr. Martindale has given, and no doubt this is the secret of the great success of his book. The index is a very full one, and its usefulness is greatly enhanced by the fact that it contains the names and the doses of many drugs not mentioned in the text, as well as those of all the pharmacopœial preparations, thus becoming a very useful posological table. If in any future edition the author wishes to find room for new drugs we would suggest that he might leave out the therapeutic index; surely no one ever uses it!

Natural Hygiene. By H. Lahmann, M.D. (London: Swan Sonnenschein and Co. 1898. Pp. 249. Price 4s. 6d.)

In this book—a translation from the German—a definite thesis is laid down. This thesis briefly is that many, if not most, of mortal ills are due to "dietetic dyscæmia." By "dietetic dyscæmia" is meant a deficiency in the body, as a result of errors of diet, of soda and lime salts. This state of "dyscæmia" is believed by the author to lead to pernicious anæmia, osteo-malacia, Graves' disease, purpura, myopia, and the cancerous diathesis. The remedy is found in the substitution of green vegetables and fruit for table salt, meat, bread, and potatos. As is usual in books of this class, much that is true is obscured by much that is fantastic. So anxious is the author to show the errors of ordinary ways and the fearful consequences of continuing in them, that he forgets that common experience shows that a use of meat and salt is only rarely attended by hæmophilia and diabetes and the rest of his dread catalogue. The author has brought his children up on his own plan, and has persuaded his wife to adopt it also. The results are detailed with much *naiveté*, and in the case of the children exemplified by photographs. The book is interesting, and deserves consideration. It is, we notice, printed in Holland; the paper employed is unworthy of the publishers' reputation in these matters.

